# Supplementary material for: Association between oral microbiome and seven types of cancers in East Asian population: a two-sample Mendelian randomization analysis
Source: Front Mol Biosci. 2023 Nov 21;10:1327893. doi: 10.3389/fmolb.2023.1327893 (PMC10702768; doi:10.3389/fmolb.2023.1327893)
Supplement: Supplementary file 1 [file DataSheet1.ZIP › Supplementary Materials/MR plots of saliva/saliva═╝/Prostate cancer/pheno.944_to_prostate cancer_leave_one_out.pdf]

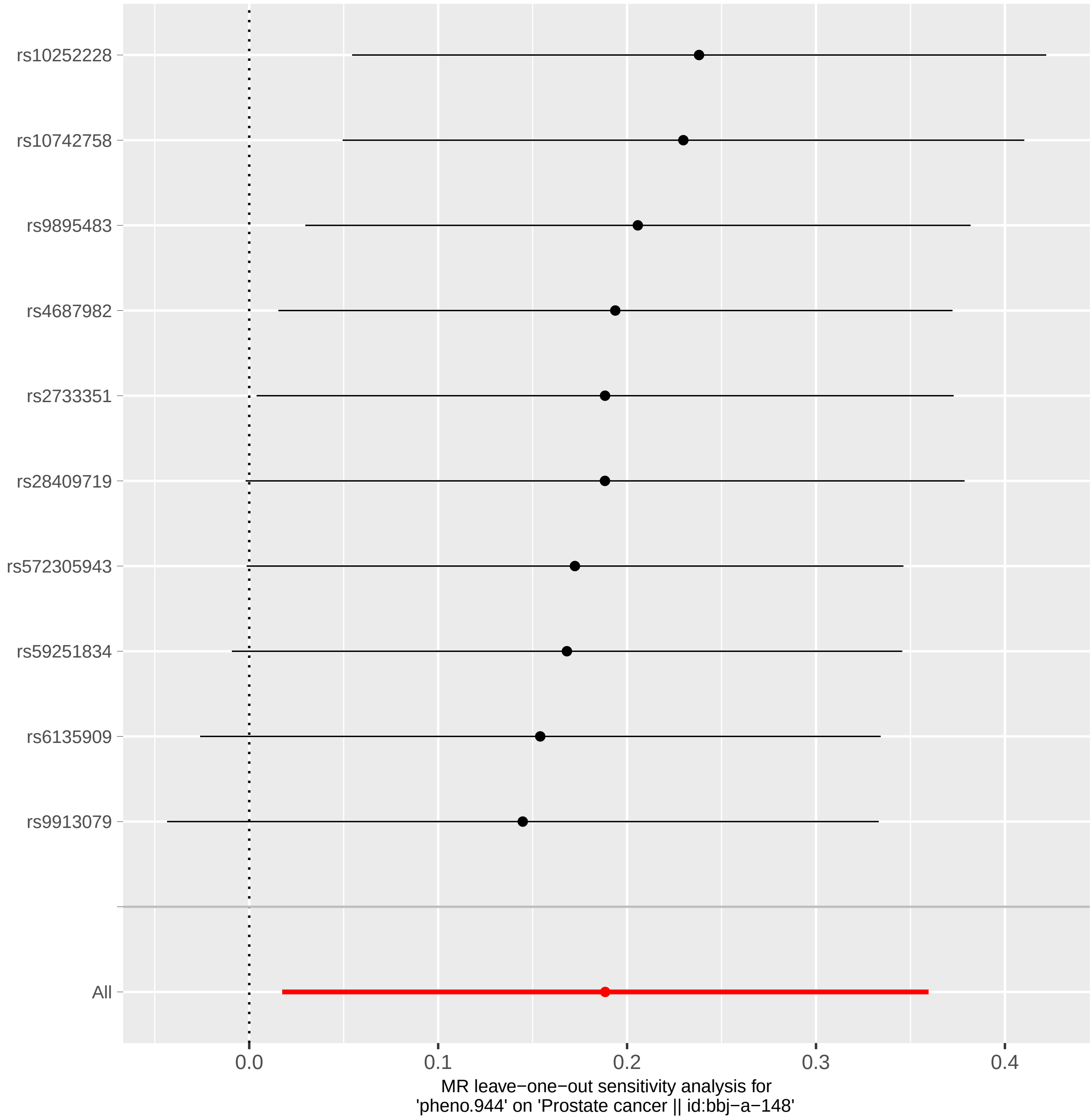
rs10252228
rs10742758
rs9895483
rs4687982
rs2733351
rs28409719
rs572305943
rs59251834
rs6135909
rs9913079
All
0.0
0.1
0.2
0.3
0.4
MR leave−one−out sensitivity analysis for
'pheno.944' on 'Prostate cancer || id:bbj−a−148'
